# Neuronal Specificity of Acupuncture in Alzheimer's Disease and Mild Cognitive Impairment Patients: A Functional MRI Study

**DOI:** 10.1155/2018/7619197

**Published:** 2018-07-17

**Authors:** Yi Shan, Jing-Juan Wang, Zhi-Qun Wang, Zhi-Lian Zhao, Mo Zhang, Jian-Yang Xu, Ying Han, Kun-Cheng Li, Jie Lu

**Affiliations:** ^1^Department of Radiology, Xuanwu Hospital, Capital Medical University, Beijing, China; ^2^Key Laboratory of Magnetic Resonance Imaging and Brain Informatics, Beijing, China; ^3^Department of Nuclear Medicine, Xuanwu Hospital, Capital Medical University, Beijing, China; ^4^Department of Radiology, Dongfang Hospital, Beijing University of Chinese Medicine, Beijing, China; ^5^Department of Integrated TCM and Western Medicine, General Hospital of Chinese People's Armed Police Forces, Beijing, China; ^6^Department of TCM, Shenzhen University General Hospital, Shenzhen, China; ^7^Department of Neurology, Xuanwu Hospital, Capital Medical University, Beijing, China

## Abstract

Although acupuncture is considered to be effective and safe for Alzheimer's disease (AD) and mild cognitive impairment (MCI), the mechanism underlying its therapeutic effect is still unknown. Most studies clarifying the neuronal pathway produced by acupuncture were still applied to healthy subjects with limited single acupuncture point stimulation, which was inconsistency with clinical practice. Thus, in our present study, we investigate the differences between brain activity changes in AD and MCI patients caused by multi-acupuncture point Siguan (four gates), in order to provide visualized evidence for neuronal specificity of clinical acupuncture. Forty-nine subjects were recruited, including 21 AD patients, 14 MCI patients, and 14 healthy controls (HC). AD and MCI patients were randomly divided into two groups, respectively: real acupuncture point group (14 AD and 8 MCI) and sham acupuncture point group (7 AD and 6 MCI). We adopted a 16-minute, single-block, experimental design for acquiring functional MRI images. We found, in AD and MCI patients, Siguan (four gates) elicited extensive activations and deactivations in cognitive-related areas, visual-related areas, the sensorimotor-related area, basal ganglia, and cerebellum. Compared with HC, AD and MCI patients showed similar activations in cognitive-related brain areas (inferior frontal gyrus, supramarginal gyrus, and rolandic operculum) as well as deactivations in cognitive-related areas, visual-related areas, basal ganglia, and cerebellum, which were not found in HC. Compared with sham acupuncture points, real acupuncture points produced more specific brain changes with both activated and deactivated brain activities in AD and MCI. The preliminary results in our study verified the objective evidence for neuronal specificity of acupuncture in AD and MCI patients.

## 1. Introduction

Alzheimer's disease (AD) is the most prevalent cause of neurodegenerative diseases characterized by progressive cognitive impairment and aging risk factor [1]. Because a dramatic shift in population demographics may occur by 2050, which is that the population older than 65 years will outnumber the population younger than 5 years by twofold to threefold, the incidence of AD, now 30 million worldwide, will triple or quadruple by then. This phenomenon will cause severe societal and economic burden to public health care due to the patients' cognitive disability and loss of independent function [2]. Mild cognitive impairment (MCI) is the clinical stage of cognition impairment between normal aging and AD patients, with a diagnostic criterion of a symptomatic, predementia phase. It is important to find early and available interventions to delay the development from MCI to dementia [3]. However, no medical treatment has been claimed to be able to stop or reverse the progression of AD or MCI. Drugs approved by the Food and Drug Administration (FDA) for AD treatment reported in mainstream medical articles were considered to have modest symptomatic effect and were always related to inevitable adverse reactions [4].

The use of acupuncture has been a major therapeutic method in traditional Chinese medicine (TCM) for treating AD over the years. A systematic review indicated that acupuncture was more effective than drugs and could, furthermore, enhance the effects of drugs in terms of improving the cognitive function of AD patients and enhancing their ability to satisfy daily needs. Moreover, the adverse reactions of acupuncture were described as tolerable and not severe, which verified the safety of clinical acupuncture application [4, 5]. Compared with drug treatments, acupuncture also appeared to produce better outcomes in MCI patients [6]. Nevertheless, the underlying mechanism of acupuncture is still unclear. Some researchers found that acupuncture may lead to beneficial brain changes in AD patients, such as ameliorating impaired cholinergic function, relieving amyloid neurotoxicity, and reducing hyperphosphorylated Tau protein and oxidative stress. However, these experimental clues were mostly controversial hypotheses, and few of them have been confirmed in vivo. Therefore, it is necessary to demonstrate the objective validation and therapeutic specificity of acupuncture before it is generally approved by clinical use [7].

Many studies held the idea that brain response to effective acupuncture therapy could be observed by functional magnetic resonance imaging (fMRI), which had provided visualized evidence for the existence of an acupuncture point–brain pathway between acupuncture stimulation and its target brain regions remote from the needling [9–12]. These findings also verified the existence of neuronal specificity in acupuncture therapy in that particular acupuncture points could produce specific brain network changes, which may reveal the mechanism underlying the neuronal modulatory effect of acupuncture. However, most research concerning acupuncture specificity was still applied to healthy subjects with various single acupuncture point stimulation locations (acupuncture with only one acupuncture point) and their sham points [11], although, in current clinical treatment decisions, multi-acupuncture point therapy (acupuncture with several combined acupuncture points) was commonly adopted, which could not only enhance the therapeutic effects but also avoid conceivable adverse reactions to acupuncture. Thus, choosing appropriate acupuncture points for specific, patient-related research needs to be seriously considered based on clinical practice.

Because of its curative effect on cognitive disorders, multi-acupuncture point Siguan (four gates) (the combination of four acupuncture points, bilateral Hegu(LI4), and Taichong(LR3)) has been used generally in AD and MCI treatment. In one of our previous studies, we demonstrated the neuronal specificity of Siguan (four gates) in healthy volunteers. We found that real acupuncture point Siguan (four gates) produced specific activations in extensive cortical and subcortical brain areas, whereas sham points only activated brain regions that were not related to specific needling locations [8]. However, whether similar findings could be extended further to apply to patients needs to be proved. Therefore, in our present study, we investigated the neuronal specificity of acupuncture in AD and MCI patients with multi-acupuncture point Siguan (four gates) by using fMRI. First, we wanted to discover the differences between acupuncture on Siguan (four gates) and its sham points in AD and MCI patients, to confirm whether real acupuncture point stimulation could produce more specific brain activity changes in cognitively impaired patients. Second, we focused on the variations between acupuncture on Siguan (four gates) in AD patients, MCI patients, and healthy people, to discover possible mechanisms underlying the neuronal modulatory effects of acupuncture.

## 2. Materials and Methods

### 2.1. Participants

Forty-nine right-handed subjects were recruited, including 21 AD patients (51–77 years old; mean age, 66.9 years old), 14 MCI patients (44–80 years old; mean age, 66.4 years old), and 14 healthy people (57–75 years old; mean age, 66.1 years old). AD and MCI patients were volunteers who had previously consulted specialist memory clinics at Xuanwu Hospital for memory complaints. Healthy controls (HC) were enrolled from the local community. All participants were informed about their basic understanding of this experiment (approved by the Medical Research Ethics Committee of Xuanwu Hospital, No. [2016]004) and gave written consent.

Before the study, all participants underwent a complete physical and neurological examination, an extensive battery of neuropsychological assessments, and standard laboratory tests. The inclusion criterion for AD patients followed the Diagnostic and Statistical Manual of Mental Disorders 4th Edition (DSM-IV) criteria for dementia and the National Institute of Neurological and Communicative Disorders and Stroke/Alzheimer Disease and Related Disorders Association (NINCDS-ADRDA) criteria for possible or probable AD [13, 14]. The inclusion criteria for MCI patients met the following standards [15]: (a) memory impairment on a normalized objective verbal memory test; (b) history of recent worsening memory symptoms; (c) Mini-Mental State Examination (MMSE) score of 24 or higher and normal or near-normal performance on other daily living scales; (d) Clinical Dementia Rating (CDR) scale of at least 0.5; and (e) not meeting the criteria for dementia. HC met the following criteria: (a) no neurological or psychiatric disorders such as stroke, depression, or epilepsy; (b) no neurological deficiencies such as visual or hearing loss; (c) no abnormal findings such as infarction or focal lesion in conventional brain MR imaging; (d) no cognitive complaints. The exclusion criteria were as follows: (a) contraindications for MRI such as pacemaker, cardiac defibrillator, implanted material with electric or magnetic system, vascular clips, or mechanical heart valve, cochlear implant, or claustrophobia; (b) history of stroke, psychiatric diseases, drug abuse, severe hypertension, systematic diseases, or intellectual disability.

**Figure 1:**
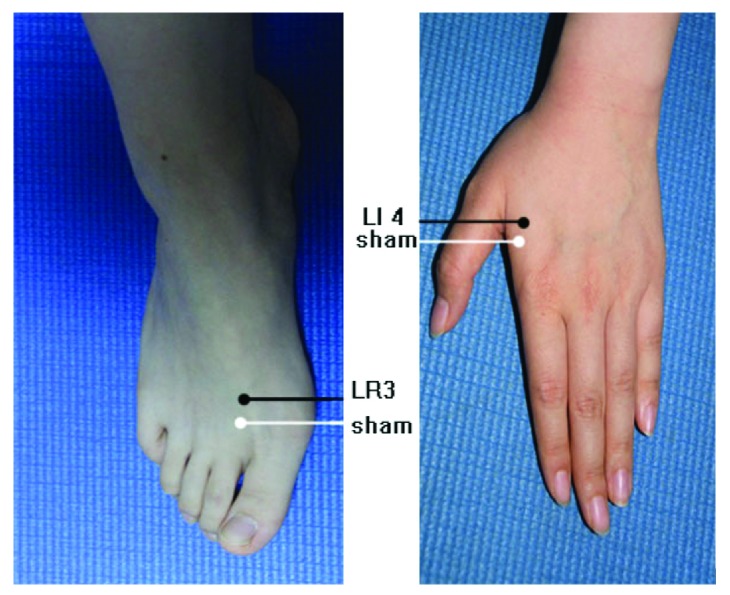
Anatomical location of real acupuncture point Siguan (four gates) and sham acupoints: Taichong (LR3), Hegu (LI4), and their sham points located 10 mm anterior to the corresponding real ones. Adapted from a Shan et al., 2014 [8].

Twenty-one AD and 14 MCI patients were randomly divided into two experimental groups, with different acupuncture stimulation procedures: real acupuncture point group (14 subjects with AD; 8 subjects with MCI) and sham acupuncture point group (7 subjects with AD; 6 subjects with MCI); 14 HC experienced only real acupuncture point stimulation. Siguan (four gates) is a multi-acupuncture point procedure that comprises four separated acupuncture points: bilateral Taichong (LR3) on the dorsum of the feet and in the depression anterior to the junction of the first and second metatarsals and bilateral Hegu (LI4) on the dorsum of the hands, at the midpoint on the radial side of the second metacarpal. The sham acupuncture points were defined as four locations approximately 10 mm next to bilateral Taichong (LR3) or Hegu (LI4). Detailed anatomical locations of Siguan (four gates) and sham acupuncture points were shown in Figure 1 (adapted from Shan et al., 2014 [8]).

### 2.2. Stimuli and Scanning Procedure

MRI data was obtained at a 3 Tesla scanner (Verio; Siemens, Erlangen, Germany). Functional images were acquired axially, using an echo-planar imaging (EPI) sequence: repetition time [TR] = 2000 ms, echo time [TE] = 40 ms, flip angle [FA] = 90°, image matrix = 64 × 64, slice number = 33, thickness = 3 mm, gap = 1 mm, and bandwidth = 2232 Hz/pixel. During the scanning procedure, subjects were instructed to stay awake, hold still, keep eyes closed, and think of nothing in particular, with hands and feet exposed for ease of acupuncture administration. We adopted a 16-minute single-block experimental design to obtain functional images. After acquiring baseline resting-state data for the initial three minutes, acupuncture stimulation was manually administered while fMRI went on scanning for the next three minutes. Stainless nonmagnetic needles of 0.3 mm diameter and 25 mm long were inserted to the depth of 2 cm into the four points (real or sham) simultaneously, with the needles rotated continuously (±180°, 60 times per minute). Finally, the needles were withdrawn, and the scan continued acquiring data for another 10 minutes to prevent the influence of unpredictable post-effect caused by acupuncture stimulation. All these manipulations were performed by the same two skilled acupuncturists in synchrony.

### 2.3. Data Analysis

In the data analysis process, fMRI data acquired during the three-minute resting-state baseline and three-minute acupuncture administration period were included. The imaging preprocessing and data analysis were performed with statistical parametric mapping software (SPM12, http://www.fil.ion.ucl.ac.uk/spm). The first four images of each session of the magnetic equilibrium were discarded. Afterwards, all functional images were preprocessed (corrected for slice acquisition time and head motion and smoothed with a Gaussian kernel of 6 mm, full width at half maximum). The head movements of all data were less than 2 mm or 2°. Then, the functional images were spatially normalized into the Montreal Neurological Institute (MNI) space with the standard EPI template. In addition, all normalized images were resliced into 3.0 × 3.0 × 3.0 mm^3^ voxels.

Based on a general linear model, the smoothed data was analyzed voxel by voxel for each individual. The reference response function was set as a boxcar waveform convolved with the Poisson Hemodynamic Response Function (HRF). Therefore, the activated or deactivated brain areas elicited by acupuncture were identified. Intra-group comparisons were performed through a one-sample* t*-test: (a) between acupuncture stimulation and baseline of the real acupuncture point group in AD patients; (b) between acupuncture stimulation and baseline of the sham acupuncture point group in AD patients; (c) between acupuncture stimulation and baseline of the real acupuncture point group in MCI patients; (d) between acupuncture stimulation and baseline of the sham acupuncture point group in MCI patients; and (e) between real acupuncture point stimulation and baseline of the healthy control group. Established by AlphaSim software, multiple comparisons were evaluated with clustering and P value criteria; the minimum cluster sizes were 17 voxels (459mm^3^), whereas the maximum P value for voxels was 0.01. All surviving voxels had an adjusted P value <0.05.

## 3. Results

### 3.1. Demographic and Clinical Neuropsychological Data

The principal demographic and clinical characteristics of participants were demonstrated in Table 1. It showed that no significant difference had been found in age and years of education among AD patients, MCI patients, and HC participants (P = 0.68, 0.60, respectively). Neuropsychological tests among AD patients, MCI patients, and HC, including Mini-Mental State Examination (MMSE), Auditory Verbal Learning Test (AVLT), and Clinical Dementia Rate (CDR) showed significant difference (P <0.01).

### 3.2. Acupuncture Stimulation at Real or Sham Acupuncture Points in AD Patients

**Figure 2:**
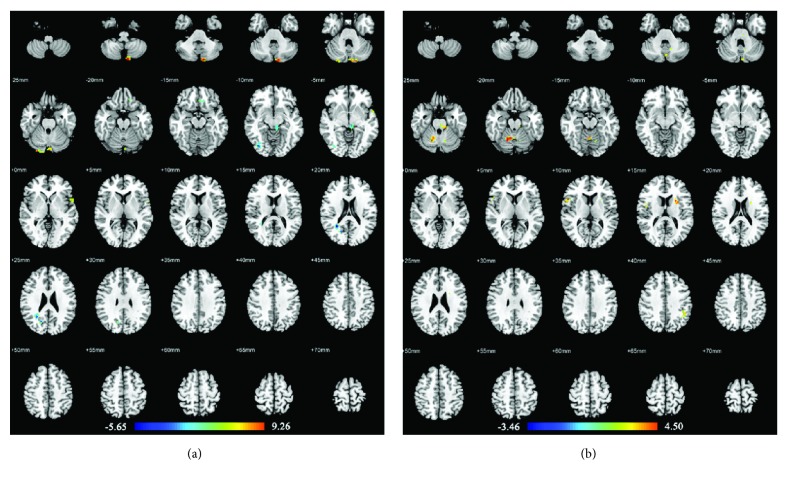
Brain regions with abnormal changes by acupuncture stimulation at real acupuncture points (a) or sham acupuncture points (b) in AD patients. Left side of the images is the right side of the brain.

In AD patients, compared with the resting-state period, acupuncture at the real acupuncture points activated brain areas primarily in the bilateral cerebellum, right inferior frontal gyrus (pars opercularis and pars orbitalis), right middle temporal gyrus, left pallidum, left rolandic operculum, left superior parietal gyrus, and left supramarginal gyrus. Decreased activations were found in the right cuneus, right pallidum, right inferior occipital gyrus, left rectus, left cerebellum, and left putamen. Alternatively, compared with the resting-state, acupuncture at the sham acupuncture points in AD patients activated brain areas primarily in the bilateral cerebellum, right rolandic operculum, and left inferior parietal angular gyrus. No decreased activities were found. For details of these brain regions, see Table 2 and Figure 2.

### 3.3. Acupuncture Stimulation at Real or Sham Acupuncture Points in MCI Patients

**Figure 3:**
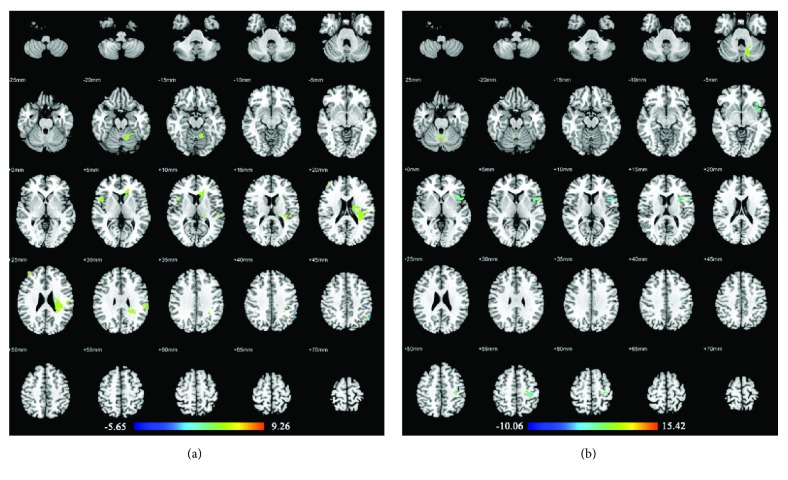
Brain regions with abnormal changes by acupuncture stimulation at real acupuncture points (a) or sham acupuncture points (b) in MCI patients. Left side of the images is the right side of the brain.

In MCI patients, compared with the resting-state period, acupuncture at the real acupuncture points activated brain areas in the left supramarginal gyrus, left superior temporal gyrus, left rolandic operculum, left cerebellum, right middle frontal gyrus, and right inferior frontal gyrus (pars opercularis). On the other hand, the brain area in the left inferior parietal gyrus (BA40) showed decreased activity. Acupuncture at the sham acupuncture points in MCI patients showed activations in the left cerebellum but decreased activities in the precentral gyrus. The details of these regions are presented in Table 3 and Figure 3.

### 3.4. Acupuncture Stimulation at Real Acupuncture Points in HC

**Figure 4:**
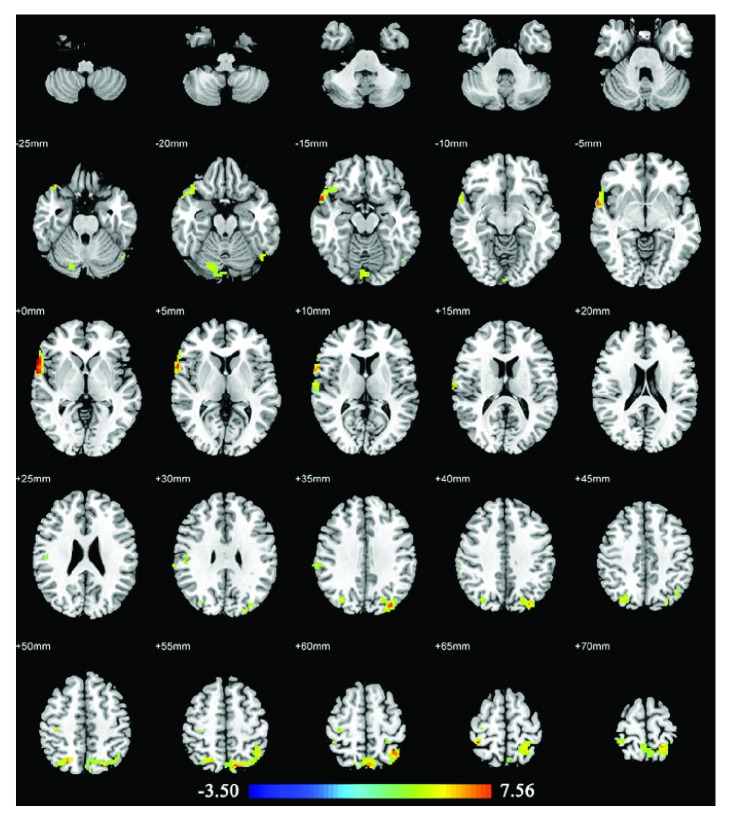
Brain regions with abnormal changes by acupuncture stimulation at real acupuncture points in healthy controls. Left side of the images is the right side of the brain.

In HC, acupuncture at the real acupuncture points activated brain areas mainly in the right superior temporal gyrus (BA22), right superior temporal gyrus, right superior parietal gyrus, right supramarginal gyrus (BA40), right postcentral gyrus, right precentral gyrus, right cerebellum, left inferior parietal gyrus, left middle occipital gyrus, and left inferior occipital gyrus. No decreased activities were found. The details of these regions are presented in Table 4 and Figure 4.

## 4. Discussion

In most clinical acupuncture point-specificity research, there are two main investigations for illustration patterns: (a) to discover the differences between acupuncture on real and sham acupuncture points and (b) to investigate the differences between acupuncture on patients and HC. Thus, in our present study, we adopted these two patterns of comparison methods to investigate the acupuncture point-specific effect of acupuncture in AD, MCI, and HC, as follows: (a) to compare the differences between acupuncture at Siguan (four gates) and its sham point in AD and MCI patients and (b) to compare the differences among AD and MCI patients and HC with acupuncture at Siguan (four gates).

### 4.1. Real versus Sham Acupuncture in AD and MCI Patients

We found that real acupuncture points elicited a wider range of increased brain activity changes than sham points. In AD patients, real acupuncture points elicited extensive activations in cognitive-related areas (inferior frontal gyrus, middle temporal gyrus, supramarginal gyrus, and rolandic operculum), the sensorimotor-related area (superior parietal gyrus), basal ganglia (pallidum), and cerebellum. In MCI patients, real acupuncture points also elicited several activations in cognitive-related areas (middle frontal gyrus, inferior frontal gyrus, superior temporal gyrus, supramarginal gyrus, and rolandic operculum) and the cerebellum. However, sham acupuncture points only activated brain areas in cognitive-related areas (inferior parietal gyrus and rolandic operculum) in AD patients, as well as the cerebellum in both AD and MCI patients. In most previous acupuncture point-specificity studies, differences in activation patterns had been generally detected between real and sham acupuncture. Real acupuncture point stimulations were often related to a wider range of activations that mainly occurred in the somatosensory area, motor area, basal ganglia, cerebellum, limbic system, and higher cognitive area [12]. Our present study showed consistency with these findings. Furthermore, we found that acupuncture on Siguan (four gates) in AD and MCI patients showed more activation in cognitive-related brain areas than in ones activated by sham acupuncture points, which may verify the therapeutic effect of Siguan (four gates) in cognitively impaired patients.

On the other hand, we found that real acupuncture points caused more deactivated changes than sham ones. In AD patients, deactivations caused by real acupuncture points were found in cognitive-related area (rectus gyrus), visual-related areas (cuneus gyrus and inferior occipital gyrus), basal ganglia (pallidum and putamen), and the cerebellum. In MCI patients, deactivation elicited by real acupuncture points was located in BA 40. Nevertheless, with sham acupuncture point stimulation, no deactivation was found in AD patients, whereas MCI patients showed only decreased activity in a sensorimotor area (precentral gyrus). These findings presented the assumption that real acupuncture points might produce both activated and deactivated neuronal modulatory patterns in AD and MCI patients, whereas sham points hardly caused deactivated ones. A systematic review reported that real acupuncture point stimulation could elicit more deactivations in the limbic system than sham ones could, when ignoring the variances of acupuncture points and subjects [12]. However, in our present study, brain areas with decreased activity elicited by real acupuncture points mainly occurred in cognitive-related areas, visual-related areas, basal ganglia, and cerebellum. This inconsistency may indicate a specific deactivated neuronal modulatory pattern of acupuncture in AD and MCI patients.

### 4.2. Patients versus HC with Real Acupuncture Stimulation

Acupuncture on Siguan (four gates) in HC elicited extensive activations in cognitive-related areas (superior temporal gyrus, inferior parietal gyrus, BA22, and BA40), sensorimotor areas (superior parietal gyrus, precentral gyrus, and postcentral gyrus), visual-related areas (middle occipital gyrus and inferior occipital gyrus), and cerebellum. Apparently, when compared with these findings in AD and MCI, HC showed the most wide-ranging brain areas that appeared to be activated changes elicited by Siguan (four gates), whereas AD and MCI patients showed fewer but more specific activations, mostly located in cognitive-related brain areas. In addition, despite some commonly activated areas (such as the cerebellum), we found activations in the right inferior frontal gyrus (pars opercularis), left supramarginal gyrus, and left rolandic operculum activation was found in both AD and MCI patients, but not in HC. The inferior frontal gyrus is part of the prefrontal associational integration, which associates with personality expression, decision making, and several other complex cognitive behaviors. The supramarginal gyrus is involved in the somatosensory association cortex with function of space and limb location, posture identification, and language production when working with an angular gyrus. The rolandic operculum in the left hemisphere is mainly related to language producing. These similarities of increased brain activities in AD and MCI patients could possibly correlate with the corresponding therapeutic effect on cognitive impairment by acupuncture at Siguan (four gates). Furthermore, we did not find any decreased activity in HC, whereas AD and MCI patients showed several specific deactivated brain areas, as mentioned earlier. These findings verified the assumption that acupuncture could produce different neuronal changes in cognitively impaired patients and healthy people.

Our current study confirmed the neuronal specificity of acupuncture that real acupuncture point stimulation administered to AD and MCI patients could produce more extensive activated as well as deactivated brain changes than sham acupuncture points. Meanwhile, we described possible modulatory patterns Siguan (four gates) elicits in cognitively impaired patients and proposed that AD and MCI patients could present similar, less, but more specific increased activities in cognitive-related brain areas than HC. Also, decreased neuronal changes were found in both AD and MCI patients, but not in HC, which showed consistency with our previous study [8]. This result suggests that decreased activity elicited by acupuncture may indicate a specific neuronal modulatory effect in AD and MCI patients. Some other studies have also reported various brain changes in AD and MCI patients after administration of acupuncture. A rat AD model proved that needling at Zusanli (ST36) could increase blood perfusion and glycol metabolism in certain brain areas [16]. In our previous studies, we also demonstrated that acupuncture could activate certain cognitive-related regions in AD and MCI patients that compared with healthy subjects [17]. In AD patients, acupuncture could modulate hippocampal connectivity [18] and impaired functional connectivity within the default mode network [19]. In MCI patients, correlations related to the temporal regions were enhanced by acupuncture at Taixi (KI3) [20], whereas brain activities elicited by Taixi (KI3) and its sham acupuncture point were different [21]. Moreover, acupuncture at Tiaoshen Yizhi acupuncture points in MCI patients presented improved connections between cognition-related regions compared with its sham points [22]. All these studies could provide objective evidence for the existence of a neuronal modulation mechanism that acupuncture produces in AD and MCI patients.

However, our study contains some controversial limitations that need to be investigated further. First, the relatively small sample size may need more subjects or repeated scans across the same subject to ensure the reliability of our results. Second, fMRI may not be the most appropriate method (although generally used nowadays) to access neuronal specificity of acupuncture because it shows only indirect MR signal measurement of blood oxygen saturation, which is not a direct demonstration of brain activity. Furthermore, some scientists emphasized validation of fMRI data analysis methods, which has to be precisely designed to decline possible false-positive results [23]. We suggest that future studies could combine fMRI with other techniques to verify the existence of neuronal specificity in acupuncture, such as electroencephalography (EEG)/magnetoencephalography (MEG) and positron emission tomography (PET).

## 5. Conclusion

In our study, we clarified neuronal specificity of acupuncture on Siguan (four gates) in AD and MCI patients. In both AD and MCI patients, we found that real acupuncture points could produce more specific brain activity changes than sham points. Furthermore, acupuncture on Siguan (four gates) in patients elicited several specific increases as well as particularly decreased activities in cognitive-related brain areas that did not appear in HC. Therefore, our findings verified objective evidence of the specific neuronal modulatory effect of acupuncture in AD and MCI patients.

## Figures and Tables

**Table 1:** Principal demographic and clinical characteristics of participants.

	**AD patients**		**MCI patients**		**HC**	**P** **value**^**∗**^
	RA	SA	RA	SA		
**Gender [M/F] (frequencies)**	14 (4/10)	7 (5/2)	8 (3/5)	6 (3/3)	14 (6/8)	
**Age [years], mean (SD)**	66.93±8.91	71.29±4.75	66.38±10.97	67.83±6.01	66.07±5.78	0.68
**Education [years], mean (SD)**	10.07±3.38	8.86±6.69	10.63±3.54	11.00±3.16	11.00±4.52	0.60
**MMSE score, mean (SD)**	15.92±4.12	11.71±7.20	25.38±1.30	25.67±2.34	28.00±1.41	<0.01
**AVLT score, mean (SD)**						
**immediate**	11.36±3.95	7.00±5.69	14.13±3.52	22.50±3.02	26.86±5.25	<0.01
**delayed**	2.64±1.60	1.57±1.62	4.38±1.60	7.83±3.92	11.07±2.76	<0.01
**recognition**	3.36±1.55	4.14±3.34	7.38±3.11	9.17±3.19	12.71±2.09	<0.01
**CDR, mean (SD)**	1–2	1–2	0.5	0.5	0	

RA, real acupuncture point group; SA, sham acupuncture point group; HC, healthy control group; MMSE, Mini-Mental State Examination; AVLT, Auditory Verbal Learning Test; immediate, immediate recall of learning verbal; delayed, delayed recall of learning verbal; recognition, recognition of learning verbal; CDR, clinical dementia rate. All plus-minus values were means ± standard deviation (SD).

^*∗*^The  P values were obtained by one-way analysis of variance tests (ANOVA) among AD patients, MCI patients, and HC.

**Table 2:** Brain regions showing abnormal activities by acupuncture stimulation (compared with the resting state) at real or sham acupuncture points in AD patients.

**Group**	**Brain Regions**	**Activity**	**BA**	**Side**	**Cluster Size**	**MNI coordinates**			**T value**
						**x**	**y**	**z**	
**AD with real acupuncture point stimulation**	Frontal_Inf_Oper	+	/	R	18	66	15	21	5.54
	Cerebellum_Crus2	+	/	L	182	-15	-90	-33	4.69
	Cerebellum_Crus1	+	/	R	34	27	-90	-27	4.87
	Frontal_Inf_Orb	+	47	R	28	57	24	-6	4.55
	Lateral Globus Pallidus	+	/	L	18	-21	-12	3	4.23
	Rolandic_Oper	+	/	L	98	-57	9	0	4.09
	Parietal_Sup	+	/	L	18	-30	-72	57	3.43
	Supramarginal	+	/	L	20	-60	-24	21	3.40
	Temporal_Mid	+	/	R	21	60	-60	0	3.15
	Cuneus	-	/	R	128	18	-69	27	-2.65
	Pallidum	-	/	R	38	21	0	-6	-2.66
	Rectus	-	/	L	45	-12	30	-18	-2.67
	Cerebellum_4_5	-	/	L	38	-12	-36	-12	-2.68
	Occipital_Inf	-	/	R	47	42	-72	-9	-2.70
	Putamen	-	/	L	24	-6	9	-6	-2.69

**AD with sham acupuncture point stimulation**	Cerebellum_4_5	+	/	R	50	9	-57	-18	4.50
	Rolandic_Oper	+	44	R	32	60	6	9	4.17
	Cerebellum_4_5	+	/	L	22	-15	-33	-24	4.21
	Cerebellum_Crus2	+	/	L	18	-9	-87	-33	4.19
	Parietal_Inf	+	/	L	19	-45	-48	39	3.95
	Cerebellum_6	+	/	L	18	-15	-63	-18	3.78
	Cerebellum_4_5	+	/	R	50	9	-57	-18	4.50

The peak voxel for each cluster and the corresponding name of the anatomical region are given. “+” represents increased activities during the period of acupuncture stimulation from resting state, whereas “-” represents decreased ones. BA: Brodmann area. The maximum P value for voxels was 0.01 and all surviving voxels had an adjusted P value <0.05.

**Table 3:** Brain regions showing abnormal activities by acupuncture stimulation (compared with the resting state) at real or sham acupuncture points in MCI patients.

Group	Brain regions	Activity	BA	Side	Cluster size	MNI coordinates			T -value
						x	y	z	
MCI with real acupuncture point stimulation	Supramarginal	+	/	L	38	-63	-30	27	9.26
	Frontal_Mid	+	/	R	20	39	51	24	5.71
	Temporal_Sup	+	/	L	17	-48	-30	12	5.36
	Rolandic_Oper	+	/	L	216	-33	-33	15	4.85
	Cerebellum_4_5	+	/	L	42	-12	-54	-15	4.80
	Frontal_Inf_Oper	+	/	R	24	51	12	6	4,50
	Parietal_Inf	-	40	L	20	-51	-60	45	-3.52

MCI with sham acupuncture point stimulation	Vermis_6	+	/	L	24	6	-57	-24	7.02
	Cerebellum_Crus1	+	/	L	35	-30	-75	-30	5.75
	Precentral	-	/	L	54	-33	-21	60	-4.04
	Precentral	-	/	L	140	-48	6	15	-4.10

The peak voxel for each cluster and the corresponding name of the anatomical region are given. “+” represents increased activities during the period of acupuncture stimulation from resting state, whereas “-” represents decreased ones. BA: Brodmann area. The maximum P value for voxels was 0.01 and all surviving voxels had an adjusted P value <0.05.

**Table 4:** Brain regions showing abnormal activities by acupuncture stimulation (compared with the resting state) at real acupuncture points in healthy controls.

Brain regions	Activity	BA	Side	Cluster size	MNI coordinates			T -value
					x	y	z	
Temporal_Pole_Sup	+	22	R	201	63	3	-3	7.04
Parietal_Inf	+	/	L	368	-42	-57	57	5.95
Occipital_Mid	+	/	L	89	-33	-81	36	5.80
Postcentral	+	/	R	26	36	-42	66	5.44
Temporal_Sup	+	/	R	28	69	-12	12	5.18
Occipital_Inf	+	/	L	24	-48	-66	-18	4.88
Parietal_Sup	+	/	R	96	24	-66	51	4.83
Precentral	+	/	R	24	33	-24	63	4.05
Supramarginal	+	40	R	36	57	-30	36	3.34
Cerebelum_6	+	/	R	77	18	-78	-21	3.88

The peak voxel for each cluster and the corresponding name of the anatomical region are given. “+” represents increased activities during the period of acupuncture stimulation from resting state. BA: Brodmann area. The maximum P value for voxels was 0.01 and all surviving voxels had an adjusted P value <0.05.

## Data Availability

The data used to support the findings of this study are available upon request from the corresponding author.
